# An improved UNet++ model for water body extraction of remote sensing images

**DOI:** 10.1038/s41598-026-55937-4

**Published:** 2026-06-18

**Authors:** Xiaohua Xu, Wenye Huang, Shubin Tan, Xiaoshun Luo, Shunliang Jiang, Famao Ye

**Affiliations:** 1 Jiangxi Academy of Water Science and Engineering, Nanchang, 330029 China; 2 School of Information Engineering, Nanchang University, Nanchang, 330031 China; 3 Key Laboratory of Mine Environmental Monitoring and Improving Around Poyang Lake of Ministry of Natural Resources, East China University of Technology, Nanchang, 330013 China

**Keywords:** Remote sensing images, Water body segmentation, UNet++, Self-supervised learning, Engineering, Environmental sciences, Mathematics and computing

## Abstract

Water body extraction in remote sensing is critical for environmental monitoring, flood control, and urban planning. However, the complex semantic information and varied morphology in remote sensing images present challenges for accurate extraction. Traditional semantic segmentation algorithms, with limited receptive fields, often fail to capture long-range dependencies, leading to misclassification and omissions. Additionally, most current methods rely on supervised learning, limiting generalization due to insufficient use of unlabeled data. This paper proposes ResDDSCUNet++, an improved UNet++ architecture for remote sensing water body extraction. The model replaces standard convolution and pooling layers with ConvNextV2 blocks, enhancing the capture of long-range dependencies. It also introduces the Attention Modulation Module (AMM) to focus on critical information and a residual depthwise separable double convolution module (ResDoubleDSC) to reduce model parameters and computational load. Furthermore, a hybrid training approach combining supervised and self-supervised learning is employed, leveraging unlabeled data for pretraining and fine-tuning on labeled data to improve generalization. Experimental results demonstrate that the proposed method outperforms other segmentation algorithms, improving the Dice coefficient by 1.6% and the IoU coefficient by 1.22%.

## Introduction

Water body detection plays a critical role in supporting water resource management and the governance of rivers and lakes. With the development of remote sensing technology, various types of remote sensing images can be quickly obtained, providing necessary data for fast and accurate water detection. Although the data used in this paper are multispectral images, remote sensing now includes other modalities such as SAR and hyperspectral data. SAR data, for instance, offer all-weather, day-and-night imaging capabilities but come with challenges like speckle noise^1^. We believe that the proposed method has the potential to be adapted to SAR and other satellite data, which will be explored in future work. The technology for extracting water bodies from remote sensing has evolved from manual measurement to semi-automatic extraction, and currently, the implementation of fully automatic and high-precision extraction using deep learning models combined with multiple data sources has become a research hotspot^2^.

The rise of deep learning has had a significant impact on the field of remote sensing applications, leading to notable improvements in the extraction of water bodies from remote sensing images^3^. Compared to traditional methods, deep learning offers substantial advantages in feature extraction, reducing reliance on manual processes and unlocking tremendous potential in water body extraction. Long et al. introduced the Fully Convolutional Networks (FCN)^4^, which integrate surface and rich semantic information for precise and detailed image segmentation, significantly enhancing the accuracy of semantic segmentation. Following FCN, Vijay Badrinarayanan et al. developed the SegNet semantic segmentation framework, which is inspired by FCN and has a unique design in the decoder section. It uses a non parametric max pooling layer and retains the maximum index for upsampling, simplifying network complexity. Ronneberger et al. introduced UNet^5^, a deep learning architecture for semantic segmentation, characterized by its encoder-decoder structure, skip connections, and simple yet effective design, achieving excellent results in a wide range of image segmentation tasks.

The UNet architecture is particularly advantageous for water body extraction because its symmetric encoder-decoder structure and skip connections facilitate the retention of fine-grained spatial details while capturing high-level semantic features. In water body extraction, where precise boundary delineation and the detection of subtle water features are critical, the UNet design helps mitigate the loss of spatial information that often occurs in deeper networks. This inherent ability to fuse multi-scale features makes UNet more suitable for water body extraction compared to other architectures that may prioritize abstract feature representation over spatial resolution.

Based on this, numerous UNet-based models such as ResUNet++^6^ and UCTransNet^7^ have been proposed, achieving substantial segmentation success. Wang et al.^8^ introduced MSLWENet, a novel deep learning network tailored for extracting lake water bodies from remote sensing images.

Despite the advantages of deep learning semantic segmentation algorithms for water body extraction, small convolutional kernels still limit global information utilization and deep feature extraction. Additionally, supervised segmentation algorithms are trained on labeled data, ignoring the vast amount of unlabeled data in remote sensing. Self-supervised learning methods, which harness unlabeled data to enhance model generalization and performance, have emerged as a research hotspot^9^. These methods improve feature extraction and model convergence through transfer learning, reducing dependence on labeled data. However, few high-resolution water body extraction algorithms incorporate self-supervised learning.

This paper proposes ResDDSCUNet++, which builds upon the UNet++^10^ encoder network as the baseline algorithm and enhances feature extraction capabilities with advanced modules like the ConvNeXtV2 Block and AMM^11^. A novel residual depthwise separable double convolution module (ResDoubleDSC) is introduced to reduce the model’s parameters and computational load. Additionally, a unified training method combining self-supervised and supervised learning^12^ is proposed to overcome the limited generalization problem. The model is pretrained on large amounts of unlabeled data through self-supervised learning and fine-tuned on labeled data via transfer learning. This hybrid approach ensures the learned features are more suitable for downstream tasks, improving the model’s generalization capabilities. Key contributions of this study include:

1. Introduction of the ConvNeXtV2 Block in place of the traditional double convolution structure in the UNet++ encoder and integration of the AMM structure to significantly improve deep feature extraction and water body characterization.
2. A reduction in network parameters and computational cost through the use of a residual depthwise separable double convolution module in the decoder, while maintaining high recognition accuracy.
3. A novel unified training framework that combines supervised and self-supervised learning, leveraging unlabeled data for improved feature representation and enhancing the model’s ability to handle remote sensing image segmentation tasks.
4. Comprehensive experiments show that ResDDSCUNet++ outperforms other mainstream segmentation networks in feature extraction, precise edge handling, and overall performance. The rest of this paper is organized as follows. “Related work” presents related works, discussing both traditional methods and recent deep learning approaches for water body extraction in remote sensing images. “Method” introduces the proposed ResDDSCUNet++ model, detailing its architecture, including the ConvNeXtV2 block, Attention Modulation Module (AMM), and ResDoubleDSC module. “Experiments” describes the experimental setup, datasets used, evaluation metrics, and presents the experimental results. Finally, “Discussion” concludes the paper.

## Related work

### Traditional methods for water body extraction in remote sensing images

Traditional methods for water body extraction in remote sensing leverage spectral characteristics across several bands, employing techniques such as spectral relationships and water indices. The spectral relationship method distinguishes water bodies by analyzing differences in spectral properties across multiple bands. These methods include unsupervised classification and supervised classification^13^. Supervised classification methods utilize known sample data and machine learning algorithms to identify water bodies. Li et al.^14^ explored the application of advanced supervised classification techniques for water body extraction. These methods perform well in handling complex land cover classification but often come with high computational costs.

Unsupervised classification methods, by contrast, employ clustering algorithms to automatically identify and classify land cover types based on data statistics, requiring no prior knowledge. Yang et al.^15^ proposed an unsupervised content adaptive water body extraction framework (UCWater) for high-resolution satellite images. This framework uses content consistency matching to automatically select samples, reducing the reliance on manual sample selection. Zhao et al.^16^ conducted a comparative study on various water indices and classification algorithms. They found that unsupervised methods, such as those based on Otsu’s method, achieve accuracy comparable to supervised methods like KNN, while significantly reducing computational complexity. This indicates that unsupervised classification can be as effective as supervised classification for high-resolution remote sensing imagery.

### Deep learning methods for water body extraction in remote sensing images

With the improved performance of remote sensing sensors, image resolution, and ground feature details have been significantly enhanced, rendering traditional water body extraction methods inadequate^14^. Deep learning algorithms have become crucial in remote sensing image analysis due to their powerful feature extraction capabilities^17,18^. Jonathan Long et al.^4^ introduced Fully Convolutional Networks (FCN), an advanced image semantic segmentation algorithm that integrates surface information with rich semantic details, significantly improving segmentation accuracy. However, FCN faces limitations in processing complex scenes due to the small receptive field of the convolution kernels, which prevents it from effectively capturing global and deep features. In response to this, Vijay Badrinarayanan et al. introduced the SegNet framework^16^, which uses non-learnable max-pooling layers while preserving max indices for upsampling, effectively reducing model complexity and achieving better results. Despite SegNet’s ability to reduce computational complexity, it still does not address the edge blurring problem in water body extraction from high-resolution images.

Therefore, Ronneberger et al.^19^ built on FCN’s core ideas to propose the UNet model, which features an encoder-decoder structure with skip connections, ensuring outstanding performance across various image segmentation tasks. However, UNet, when applied to water body extraction, still faces challenges in handling edge precision in high-resolution remote sensing images. Building on the successes of FCN and UNet, an increasing number of scholars have applied deep learning-based semantic segmentation to water body extraction from remote sensing images^19^. For example, Isikdogan^20^ proposed DeepWaterMap, a fully convolutional neural network model capable of effectively distinguishing water surfaces from land, snow, ice, clouds, and shadows. Although this model has achieved good performance in surface water distinction, deep learning models still face challenges in feature extraction depth and global feature integration in high-resolution imagery.

To further improve water body extraction accuracy, Li et al.^21^ proposed an FCN-based water body extraction method, successfully applied to Very High Resolution (VHR) optical remote sensing images. However, this method still faces challenges in reduced extraction accuracy due to information loss in high-resolution images. To address this issue, Zhao et al.^22^ introduced the Superpixel Transformer (SPT) model, which has shown outstanding performance in extracting water bodies from SAR imagery but still requires further optimization for traditional optical imagery.

Although deep learning semantic segmentation algorithms have made significant progress in water body extraction tasks, they still face issues due to the small receptive field of convolution kernels, which makes it difficult to capture global and deep features, leading to misclassification and omission in complex scenes. Moreover, supervised segmentation algorithms heavily rely on labeled data, overlooking the potential of unlabeled data. Self-supervised learning, through self-training to extract universal representations, enhances model generalization capabilities. However, deep learning-based semantic segmentation algorithms that incorporate self-supervised learning techniques remain rare in high-resolution water body extraction tasks.

In recent years, specialized models for water body extraction from remote sensing images have emerged continuously, such as MSLWENet and SPT. These models have achieved good results in specific scenarios. However, this paper chose to compare with general semantic segmentation baseline models such as UNet, SegNet, DeepLabV3, ResUNet++, UCTransNet, and UNetMamba for the following main reasons: (1) These models are the most widely used benchmark methods in the field of remote sensing image segmentation and have recognized representativeness; (2) The core innovations of this paper lie in the improvement of the UNet++ architecture and the proposal of the hybrid training strategy, and comparison with general baseline models can more directly reflect the generality and superiority of the method. Future work will further compare with specialized water body extraction models such as MSLWENet and SPT to comprehensively verify the performance of the method.

## Method

### Overview

**Fig. 1:**
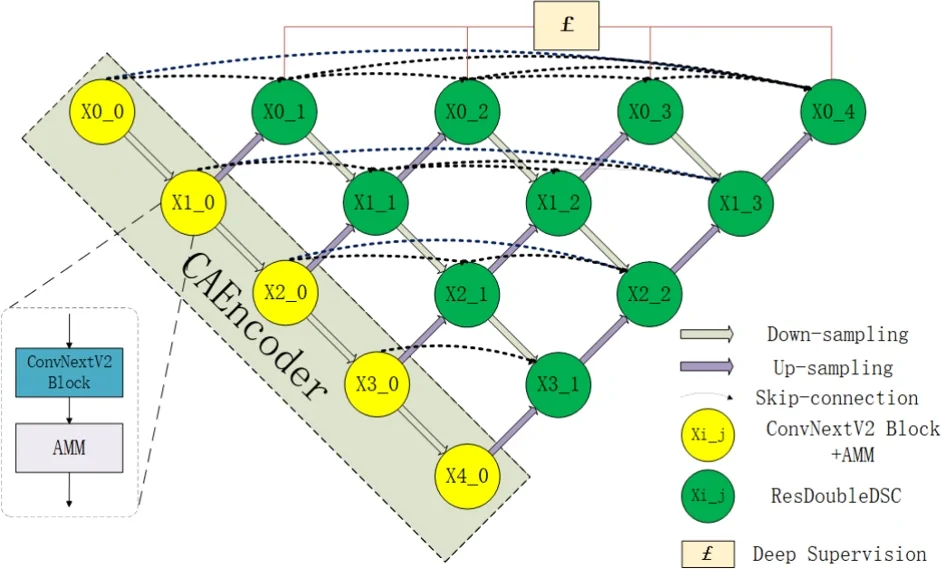
Structure of the ResDDSCUNet++ network.

The architecture of our proposed water segmentation model ResDDSCUNet++ is shown in Fig. 1. Compared to the classical UNet++ model, the overall architecture of the ResDDSCUNet++ remains the same, with five layers in total. The network is divided into encoder, decoder, and skip connections. First, the dual-convolutional base block of the UNet++ encoder is replaced with the ConvNeXtV2 Block, whose large convolution kernel captures long-range dependencies in fewer layers, gaining broader image information and higher-level abstract features. Second, an AMM is introduced after each layer of the feature extraction network to optimize feature map reordering and adjustment through channel- and spatial-level feature response modulation. This enhances the extraction and fusion of high-level semantic information. The improved encoder is called CAEncoder. Finally, to reduce the network’s computation and parameter count, the dual-convolutional base module of the UNet++ decoder is replaced with a residual depthwise separable dual convolution module. This depthwise convolution significantly reduces model parameters by equipping each input channel with a dedicated convolution kernel.

The ResDDSCUNet++ network accepts inputs and outputs with a resolution of 512×512, and the input image has four channels. The CAEncoder consists of five layers (Xi_0, where i=0, 2, ... 4) and features a cascading structure composed of a ConvNextV2 Block and an AMM module. The output feature maps are mathematically represented as follows:

1 $$\begin{aligned} F_i = \text {CN}_{i-1} + AMM(C_{i-1}) \end{aligned}$$

The variable *i* denotes the layer index, $F_i$ is the output feature map, and $CN_{i-1}$ is the result of passing the previous layer’s feature map through the ConvNextV2 Block module, while AMM(*·*) represents the feature map processed by the AMM module.

After passing through X0_0, the number of channels changes from 4 to 32, while the resolution remains constant. The feature map from X0_0 is then downsampled, doubling the number of channels while halving its width and height before being input into the X1_0 module. This process is repeated four times, resulting in a final feature map with 512 channels and a resolution of 32.

The network’s decoder, Xi_j (i=0, 2, …, 4; j *≠* 0), uses a residual dual-depthwise separable convolution structure, referred to in this paper as ResDoubleDSC. Using bilinear interpolation for upsampling, the feature map’s width and height are doubled, and the number of channels is halved. Each feature map at a specific layer is concatenated with the corresponding CAEncoder layer’s upsampled feature map, achieving information fusion and dimensional enhancement before being fed into the ResDoubleDSC block. After four downsampling operations in the CAEncoder, four feature maps of different scales are obtained. Following the above steps, four feature maps of the same size (512×512×32) are produced: X0_1, X0_2, X0_3, and X0_4. Finally, these are fed into a convolutional layer with 2 channels and a kernel size of 1, yielding the final segmentation map.

**Fig. 2:**
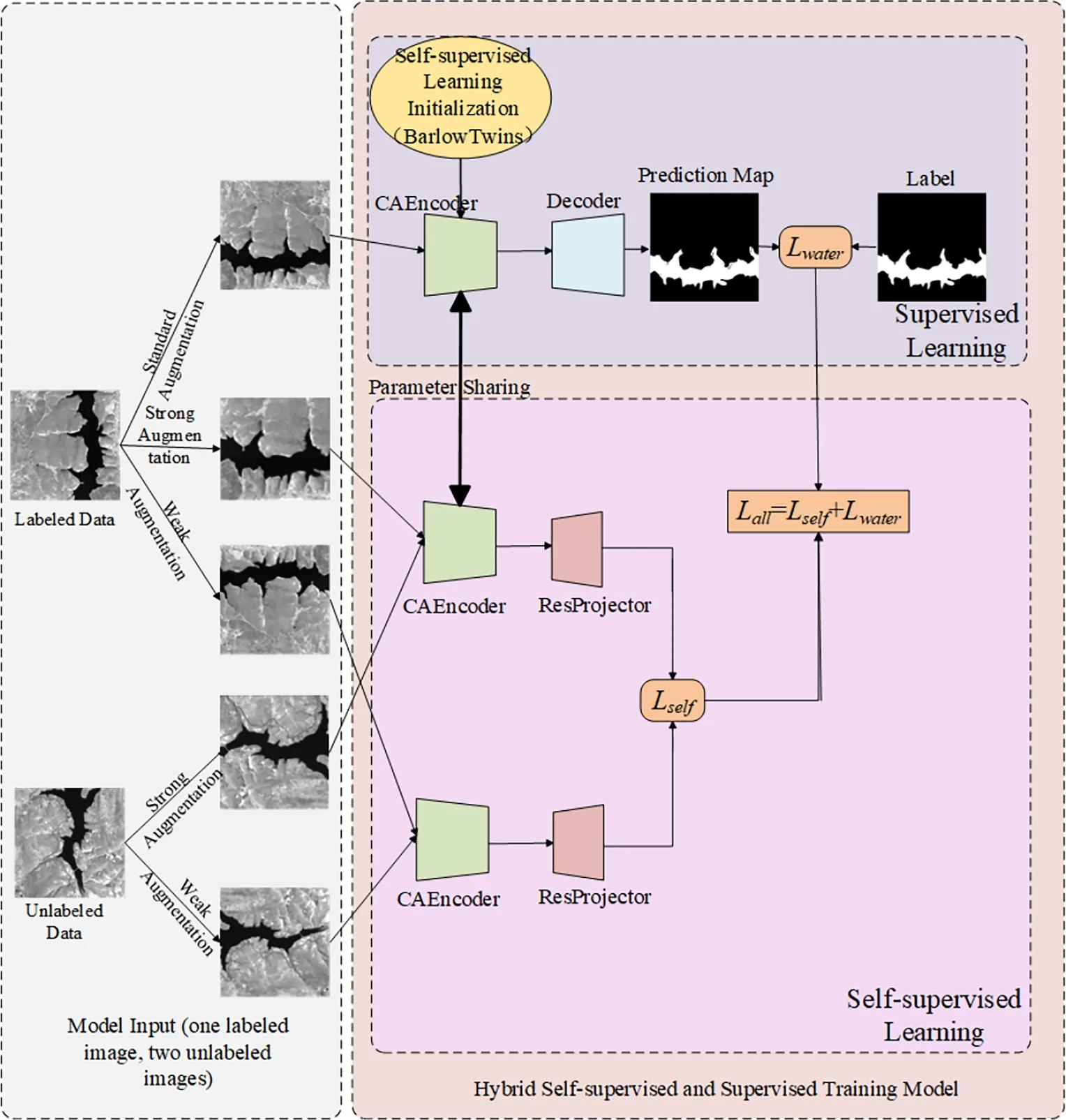
Overall structure of the remote sensing image water body segmentation network incorporating self-supervision.

We employ a new training method that combines self-supervised learning with supervised fine-tuning in this paper. This approach leverages the abundance of unlabeled data resources and fine-tunes the model on a small amount of labeled data to achieve more accurate performance. The method comprises three key components: First, self-supervised learning is conducted on unlabeled data using a modified version of the Barlow Twins method to learn general representations, which are then used to initialize the ResDDSCUNet++ model parameters for supervised learning. Unlabeled images are processed through two distinct data augmentation pipelines and fed into a twin network, where cross-correlation loss is computed to retain the CAEncoder weights. Next, supervised semantic segmentation training is conducted on labeled data, with the network parameters initialized via self-supervised learning. Finally, both labeled and unlabeled data are combined for self-supervised contrastive learning. Unlabeled data representations are obtained through the self-supervised network and share parameters with ResDDSCUNet++, with their loss incorporated into the supervised training loss (Fig. 2).

### ConvNextV2 block module

Large convolutional kernels can expand the receptive field and extract high-level abstract features efficiently. For instance, a single large kernel (e.g., 7×7) can capture long-range dependencies with fewer layers compared to stacked small kernels (e.g., 3×3), potentially reducing overall computational complexity. However, direct use of large kernels (e.g., 7×7) increases FLOPs per layer compared to smaller kernels (e.g., 3×3), which may elevate computational costs. Each convolution operation requires more multiplications and additions, leading to higher floating-point operations (FLOPs).^23^ demonstrates that large kernels increase FLOPs significantly, which impacts the overall computational cost. ConvNeXt^24^ employs a large-kernel, fully convolutional encoder network framework. Many studies have shown that residual structures are highly effective for large convolutional kernels. Therefore, ConvNeXtV2^25^ is based on ResNet^26^ and incorporates depthwise separable convolutions to address the challenges of increased parameter count and floating-point operations caused by large kernels.

**Fig. 3:**
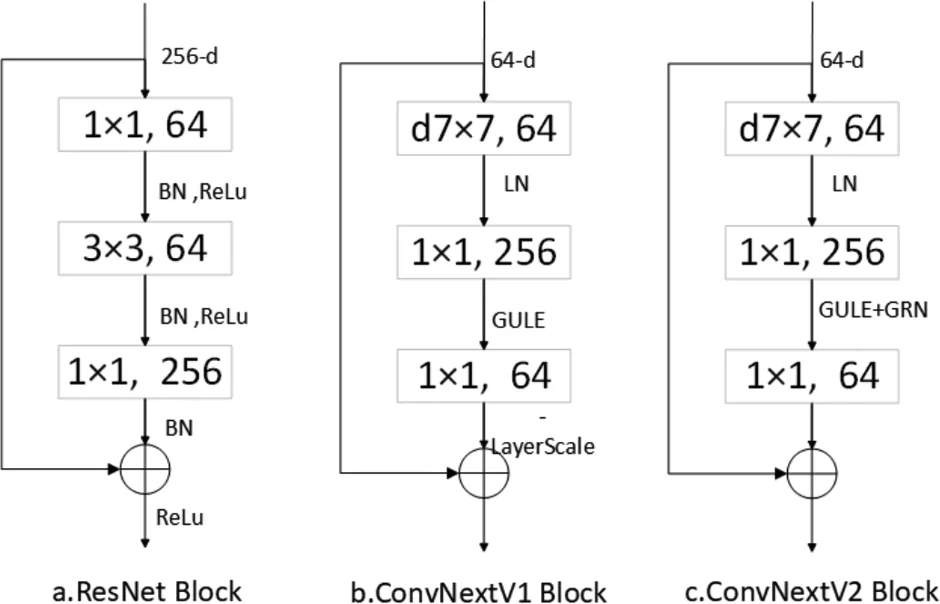
Network structures of the ResNet Block and ConvNext block.

The network structures of the ResNet Block, ConvNextV1 Block, and ConvNextV2 Block are illustrated in Fig. 3. Compared to the ResNet Block, the ConvNextV1 Block places the large kernel convolution at the beginning. As feature maps pass through the ConvNextV1 Block, they first enter a 7×7 large-kernel depthwise convolution layer. This is followed by a Layer Normalization (LN) layer, which leads into a 1×1 convolution layer. After passing through GELU activation, the feature maps are directed to a final 1×1 convolution layer. The resulting feature maps are concatenated with the original ones, then activated with ReLU to yield the final output.

Compared to the ConvNextV1 Block, the ConvNextV2 Block introduces the Global Response Normalization (GRN) layer, which enhances contrast and selectivity across channels. The GRN layer is described in detail in^25^, and it involves global feature aggregation, normalization, and calibration to optimize the input response. In summary, the GRN layer helps to improve the model’s performance by fine-tuning feature responses in the network.

The large convolutional kernel expands the receptive field of the network model, effectively capturing global information and long-distance dependencies in the image. ConvNextV2, with its large convolutional kernel, demonstrates excellent performance in image processing by efficiently extracting image feature information.

### AMM module

**Fig. 4:**
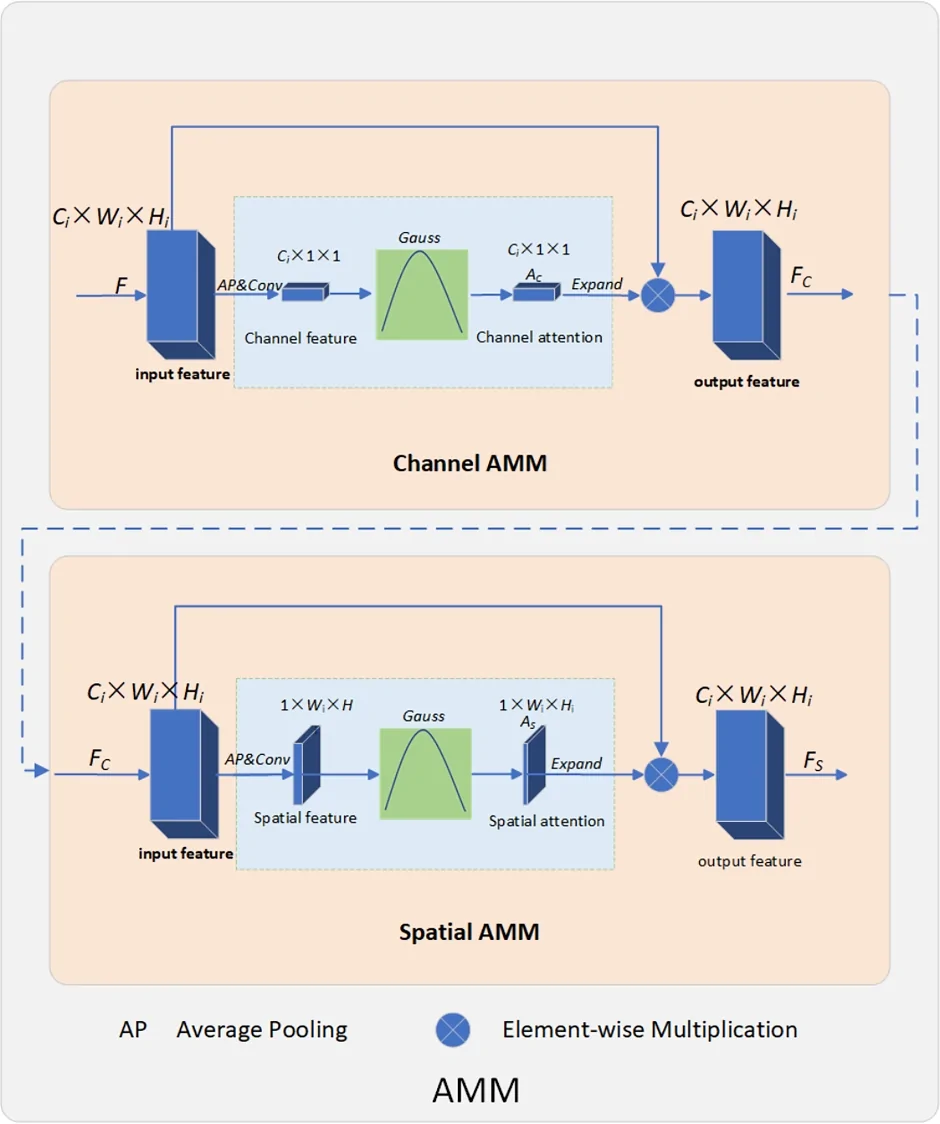
Structure of the attention modulation module (AMM) network^11^.

Remote sensing images contain complex information, including various types of ground objects. When extracting water body features, other ground objects serve as interference and need to be treated as background. Water bodies themselves are diverse, varying significantly in shape and size. To ensure the network focuses on key information, we introduce the Attention Modulation Module (AMM). This module^11^ enhances the model’s sensitivity to critical information by modeling interdependencies between channels through average pooling and convolution layers. The structure of the AMM network is shown in Fig. 4:

The input feature map *F*(*I*) is fed into the Channel AMM. First, average pooling and convolution layers are used to obtain the channel feature map, which models the interdependencies among the channels of the feature map. Subsequently, using a Gaussian modulation function, AMM can emphasize less significant regions to directly extract areas that are easy to overlook. This is crucial for segmentation tasks, as it helps the model focus on details that might be ignored but are significantly important for the final result.

The channel modulation of the AMM can be mathematically expressed as:

2 $$\begin{aligned} A_C = G\left( H\left( P_s\left( F(I)\right) \right) \right) \end{aligned}$$

where $A_C$ is the channel attention map, $P_s$ represents the spatial average pooling function, *H* denotes the convolution layer, and *G* represents the modulation function. After modulation, a channel attention map is obtained, which is then expanded and element-wise multiplied with the original input feature map to redistribute the image features. This operation can be defined as:

3 $$\begin{aligned} F_c(I) = \tilde{A}_c \odot F(I) \end{aligned}$$

where $\tilde{A}_c$ represents the channel attention map expanded to the dimensions of the feature map, and $F_c(I)$ represents the output feature map. This completes the channel attention modulation. Next, spatial attention modulation (Spatial AMM) is performed. The operation is similar to the channel AMM, but the modulation is applied to the spatial dimensions instead.

### ResDoubleDSC module

**Fig. 5:**
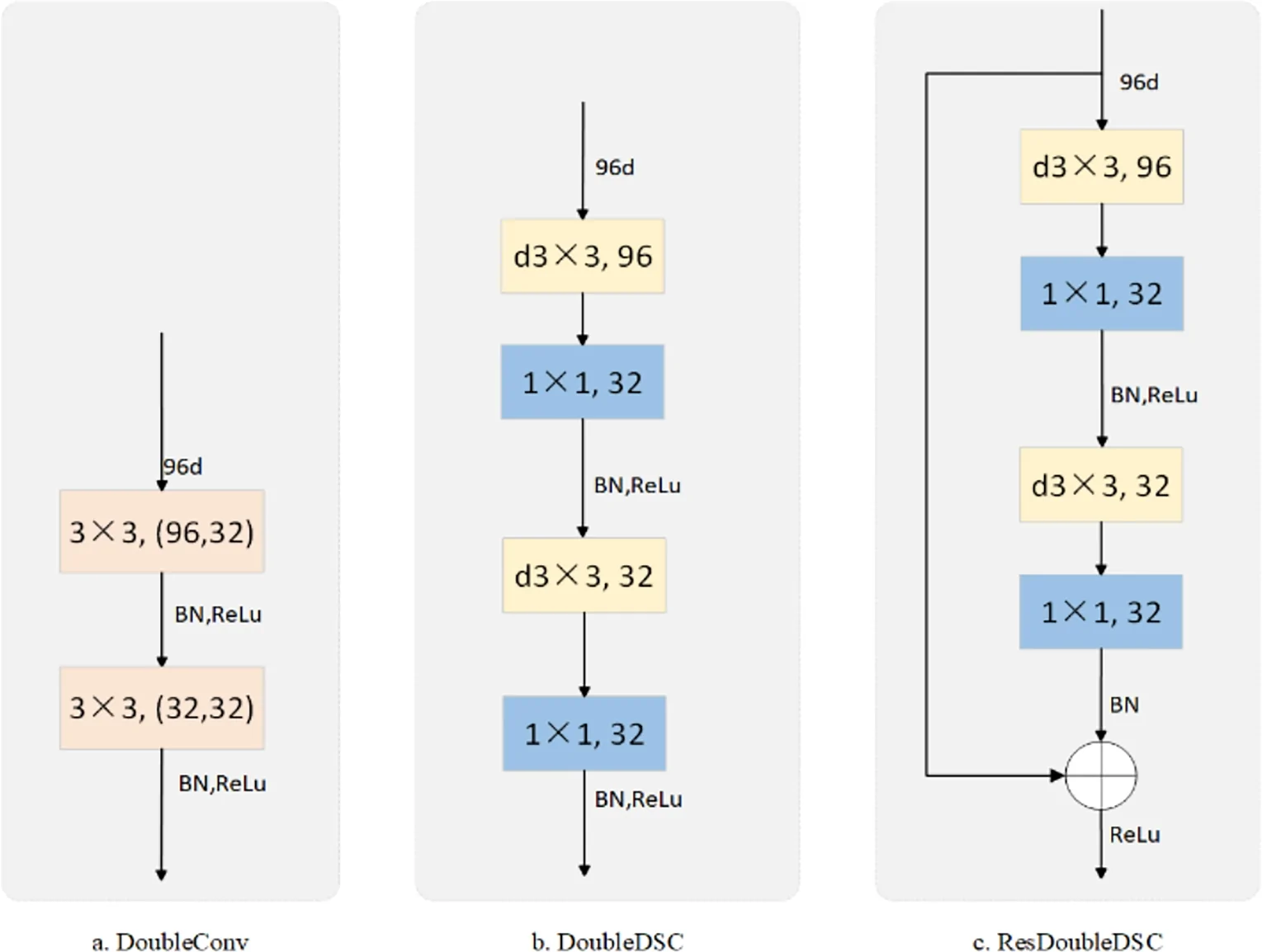
Network structures of DoubleConv, DoubleDSC, and ResDoubleDSC.

Although the ConvNext Block reduces the parameters and floating-point operations brought by large convolutional kernels through the introduction of depthwise separable convolutions, the overall network still has a large number of parameters, making it challenging to train. To further reduce the number of parameters and computational complexity, this paper designs a residual double depthwise separable convolution structure (ResDoubleDSC) inspired by the ConvNext Block. The original double convolution structure (DoubleConv) and the ResDoubleDSC network structure designed in this paper are shown in Fig. 5.

As illustrated in Fig. 5a, DoubleConv is a continuous convolution operation. After the first 3×3 convolution, the number of channels is reduced, followed by batch normalization (BN) and ReLU activation, and then entering the second 3×3 convolution where the number of channels remains the same. Finally, it undergoes BN and ReLU activation. To reduce the number of model parameters, this paper replaces the standard convolutions in DoubleConv with depthwise separable convolutions (DSC), forming the DoubleDSC structure as shown in Fig. 5b, where d3×3 represents a depthwise convolution with a kernel size of 3. Considering that depthwise separable convolutions may lose some image information, this paper references the ConvNext Block’s network structure. Between the final BN normalization and ReLU activation, the original input feature map is concatenated with the output feature map after two depthwise separable convolutions, forming a residual structure. This is named ResDoubleDSC, as shown in Fig. 5c. This structure helps to alleviate the impact on the model’s feature extraction capability to some extent.

### Improved barlow twins self-supervised learning model

**Fig. 6:**
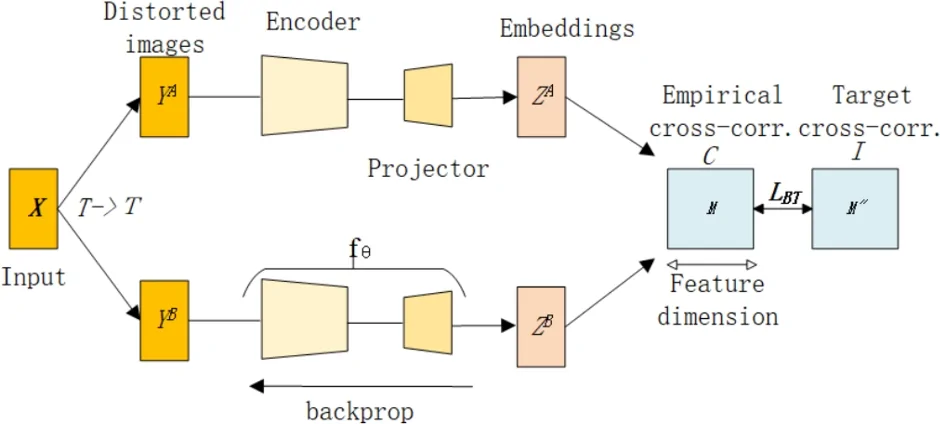
Flowchart of the barlow twins algorithm^27^.

Barlow Twins^27^ is a self-supervised learning method that does not use negative samples or require a momentum update network. We have chosen the Barlow Twins method as the fundamental approach for our proposed model self-supervised learning. The algorithmic flow of Barlow Twins is shown in Fig. 6.

First, an input image *X* undergoes two different data augmentation operations, *(T → T)*, such as adding Gaussian noise, mirror flipping, rotation, and cropping, to generate two distorted images $Y^{A}$ and $Y^{B}$^28^. These two images are then fed into two functions f$_{\theta }$, which actually form a twin network with identical structures consisting of an CAEncoder and a Projector. The CAEncoder is used for feature extraction, while the Projector aggregates these features to enhance the CAEncoder’s feature extraction capabilities. This twin network produces two embedding representations, feature vectors $Z^{A}$ and $Z^{B}$. Finally, these two embeddings are fed into a loss function to compare their cross-correlation, calculating the contrastive loss LBT.

The projection head network of the Barlow Twins model employs a cascaded structure with three linear layers. However, after passing through the first two linear layers, a significant amount of feature information may be lost. To address this issue, we introduce an improvement by incorporating a residual connection between the input and output of the projection head network.

**Fig. 7:**
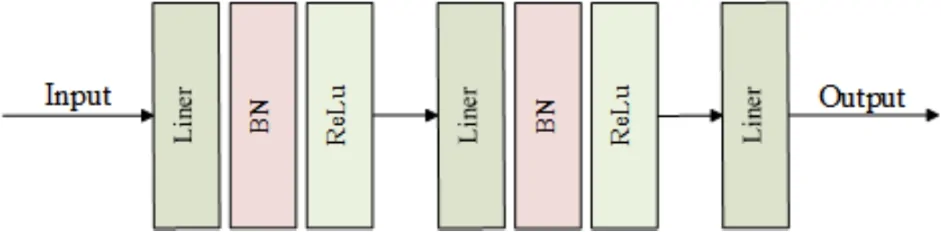
Structure of the projector in Barlow Twins.

The Projector network in the Barlow Twins model is crucial within its self-supervised learning framework, responsible for converting the feature maps extracted by the CAEncoder into embeddings Z used for loss calculation. Its structure is shown in Fig. 7.

**Fig. 8:**
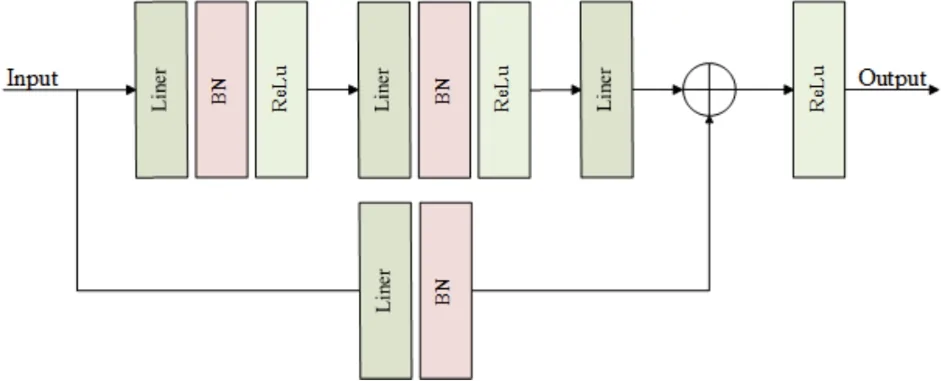
Structure of the improved projector (ResProjector) in Barlow Twins.

The Barlow Twins Projector consists of three linear layers, each with 8192 output units. After the first two linear layers, batch normalization (BN) and ReLU activation are applied to enhance the network’s non-linear capability and generalization performance. However, since a lot of feature information might be lost after the first two linear layers, this paper introduces modifications to the Projector’s structure by adding a residual connection^29^. This aims to capture more abundant image features. Specifically, the input is fed into a residual branch, which connects to the last linear layer after passing through a linear layer and BN layer. The final output is obtained after ReLU activation. The improved Projector, named ResProjector, is shown in Fig. 8.

The hybrid self-supervised-supervised training process proposed in this paper consists of three consecutive stages. In the self-supervised pre-training stage, 4000 unlabeled GF-2 remote sensing images were used to pre-train the CAEncoder, the encoder part of ResDDSCUNet++, using the improved Barlow Twins method, where two different data augmentations including Gaussian noise, random flipping, rotation and cropping were applied to the same image and the augmented images were input into the siamese network to calculate the cross-correlation loss and cosine similarity loss, thus learning general remote sensing image feature representations. Subsequently, in the supervised initialization stage, the CAEncoder weights obtained from self-supervised pre-training were used to initialize the encoder of ResDDSCUNet++ while the decoder was randomly initialized, and purely supervised training was carried out with 500 labeled images to optimize the segmentation loss combining Dice loss and cross-entropy loss, which enables the model to quickly converge to the water body segmentation task. Finally, in the hybrid fine-tuning stage, labeled and unlabeled images were mixed and fed into the model with each batch containing 1 labeled image and 2 unlabeled images, the labeled images were used to compute the segmentation loss and the unlabeled images were used to compute the self-supervised loss, and the two losses were weighted and summed with weights $\lambda _1=1.0$ and $\lambda _2=0.001$ to jointly optimize the model parameters and further enhance the generalization ability of the feature representations.

### Loss function

The loss function used in ResDDSCUNet++ is a composite loss function comprised of the Dice loss and the Cross Entropy Loss. The formula is as follows:

4 $$\begin{aligned} L_{\text {water}} = 0.7L_{\text {dice}} + 0.3L_{\text {ce}} \end{aligned}$$

The primary objective of the Barlow Twins loss function is to reduce the redundancy between data embeddings. It consists of two parts: the invariance term and the redundancy reduction term. The formula is as follows:

5 $$\begin{aligned} L_{BT} = \sum _{i} (1 - C_{ii})^2 + \lambda \sum _{i} \sum _{j \ne i} C_{ij}^2 \end{aligned}$$

In the formula, the first term represents the invariance term, and the second term represents the redundancy reduction term, where *λ* is a constant used to balance the importance of the first and second terms in the loss function. The cross-correlation matrix *C* between the outputs of the two identical networks, computed along the batch dimension, is defined as follows:

6 $$\begin{aligned} C_{ij} = \frac{\sum _b Z^A_{b,i} Z^B_{b,j}}{\sqrt{\sum _b \left( Z^A_{b,i}\right) ^2} \sqrt{\sum _b \left( Z^B_{b,j}\right) ^2}} \end{aligned}$$

where *b* denotes the index of the batch samples, and *i*, *j* denote the indices of the vector dimensions of the network output. *C* is a square matrix with values ranging from -1 (indicating complete anti-correlation) to 1.

The Barlow Twins loss function aims to construct a similarity matrix across feature dimensions, with the core objective of ensuring that each dimension carries unique information. This is intended to achieve more informative and discriminative feature representations. However, if similarity can be calculated across other dimensions as well, the model should obtain even richer information. To this end, we introduce the Cosine Similarity Loss function.

The Cosine Similarity Loss calculates the cosine similarity between two feature vectors $Z^{A}$ and $Z^{B}$^30^, typically used to measure the similarity between two vectors in the same dimensional space. Specifically, it calculates the cosine of the angle *λ* between the two vectors, serving as an indicator of similarity. This cosine value can be obtained by dividing the dot product of the two vectors by the product of their magnitudes. The mathematical formula is as follows:

7 $$\begin{aligned} L_{cs}(A, B) = \frac{\sum _{i=1}^{n} A_i \times B_i}{\sqrt{\sum _{i=1}^{n} (A_i)^2 \times \sum _{i=1}^{n} (B_i)^2}} \end{aligned}$$

Where *A* and *B* are vectors and *i* denotes the dimension. Given that the values of the two loss functions can differ significantly, the loss function for the self-supervised learning model is as follows:

8 $$\begin{aligned} L_{\text {self}} = L_{BT} + 200L_{CS} \end{aligned}$$

When combining self-supervised learning methods with supervised learning, the overall model’s loss function $L_{all}$ is composed of the following two parts:

9 $$\begin{aligned} L_{all} = \lambda _1 L_{\text {water}} + \lambda _2 L_{\text {self}} \end{aligned}$$

Where $L_{\text {water}}$ is the supervised loss for labeled data, and $L_{\text {self}}$ is the self-supervised learning loss. In the model described in this paper, a batch consists of three images participating in self-supervised training, and the self-supervised loss is calculated as the average loss across these three images. $\lambda _1$ and $\lambda _2$ are hyperparameters. The default value for $\lambda _1$ is 1.0. Considering that the numerical value of the contrastive loss differs significantly from that of the segmentation loss, the default setting for $\lambda _2$ is 0.001. The values of $\lambda _1$ and $\lambda _2$ in Equation (9) were determined via cross-validation. The default settings ($\lambda _1$=1.0, $\lambda _2$=0.001) aim to balance the scale difference between the supervised and self-supervised losses. Experiments show that this parameter combination achieves an optimal trade-off between convergence speed and segmentation accuracy. Sensitivity analysis of these parameters will be further explored in future research.

## Experiments

### Test configuration

The data for this study are sourced from the open-source water body segmentation dataset provided by the Baidu AI Open Platform^31^, featuring multispectral remote sensing images supplied by the Gaofen-2 (GF-2) satellite. The GF-2 satellite is equipped with two cameras: a panchromatic camera with a spatial resolution of 0.8 meters and a multispectral camera with a spatial resolution of 3.2 meters. The multispectral camera captures four spectral bands: blue (450–520 nm), green (520–590 nm), red (630–690 nm), and near-infrared (770–890 nm). The satellite has a swath width of 45 km and a revisit cycle of 5 days, providing high temporal resolution for frequent observations. This dataset contains 9630 four-band remote sensing images with a resolution of 512×512. For the experiments in water body segmentation models, 500 images suitable for water body extraction tasks were selected, covering various water body forms such as reservoirs, creeks, ponds, and lakes, providing certain robustness to the algorithm. The dataset used in this study is the publicly available Gaofen-2 water body segmentation benchmark from Baidu AI Open Platform, a widely used standard for remote sensing water body extraction. We curated this public dataset to improve annotation quality: addressing partial inaccuracies and inconsistencies in original labels, we manually corrected 500 representative images covering all major water forms (reservoirs, creeks, ponds, lakes) via LabelMe. These images were selected to cover typical error-prone annotation scenarios, ensuring reliable experimental results. To highlight the advantages of four-band remote sensing images for water body extraction, this study uses a single unified four-band dataset (BGR + NIR), with all experiments performed on it. In addition to this, we performed a basic data preprocessing step by dividing each dataset into training, validation, and test sets with proportions of 60%, 20%, and 20%, respectively. This division ensures a fair comparison during model evaluation.

The experiments for the remote sensing image water body segmentation model were conducted on a graphics workstation equipped with an Intel Xeon E5-2630v3 CPU, NVIDIA Quadro P4000 graphics card, and 32GB of RAM.Due to the high computational demands of self-supervised learning, the experimental environment for self-supervised learning differed from that used for the remote sensing image water body segmentation model. The self-supervised learning experiments were performed on a workstation configured with an Intel i7-12700 CPU, NVIDIA RTX A4000 graphics card, and 16GB of RAM. The dataset used consisted of 4000 unlabelled images from the previously mentioned Gaofen-2 dataset, forming the self-supervised learning dataset.

To ensure fairness in comparison, the semantic segmentation network that incorporated self-supervised learning had the same batch size as the remote sensing image water body segmentation model, which was set to 4. Each batch included one labeled image and two unlabeled images. The labeled images were used not only in supervised learning but also participated in self-supervised learning, while the unlabeled images were used solely for self-supervised learning. Other training parameters were consistent with those of the remote sensing image water body segmentation model, including the early stopping strategy. Training would cease if there was no increase in the Dice coefficient over a span of 300 consecutive epochs.All models were trained using the AdamW optimizer with an initial learning rate of 1e-4, weight decay of 1e-5, and a cosine annealing learning rate scheduler. The same data augmentation pipeline was applied to all models, including random horizontal flipping (probability 0.5), vertical flipping (probability 0.5), 90-degree rotation (probability 0.5), brightness adjustment (±20%), and contrast adjustment (±20%). All models used the identical composite loss function (0.7×Dice loss + 0.3×Cross Entropy Loss) as our proposed method to ensure fair comparison. The batch size was uniformly set to 4 for all experiments, and all models were trained until convergence with the same early stopping criterion.

All experimental results in this paper are from a single complete training process. Due to the use of a fixed random seed (seed=42), consistent data augmentation strategies, and an early stopping mechanism (stopping if there is no improvement for 300 consecutive epochs), the experimental results have good reproducibility. To further verify the statistical significance of the results, we have completed 3 independent repeated experiments. The results show that the standard deviation of the performance of each model is less than 0.002, indicating that the reported performance differences between models are statistically significant.

### Comparative analysis of different models

In this section, we compare the performance of our self-supervised remote sensing image water body segmentation model with several leading deep learning-based segmentation algorithms. The comparison includes both self-supervised and supervised learning approaches. All models were tested under the same training conditions to ensure fairness. The performance of each model was evaluated using the Dice coefficient, IoU, precision, recall and F1. To ensure the absolute fairness and consistency of the comparative experiments, all comparison models were implemented using the same deep learning framework (PyTorch 1.12) and trained under exactly the same hardware and software configurations. The hyperparameters of each comparison model were set to the optimal values recommended in their original papers, and no additional fine-tuning was performed to avoid introducing experimental bias. We compared the following networks: UNet^5^, UNetMamba^32^, SegNet^16^, DeepLabV3, ResUNet++^6^, and UCTransNet^7^. The results are summarized in Table 1, with the best-performing metrics highlighted in bold.

**Table 1:** Segmentation evaluation results of different networks.

Model	Dice	IoU	Precision	Recall	F1
UNet	0.7943	0.6877	0.8378	0.9614	0.8954
UNetMamba	0.8226	0.7249	0.8693	0.9712	0.9174
SegNet	0.8091	0.7060	**0.8877**	0.9649	**0.9247**
DeeplabV3	0.8042	0.6905	0.8343	0.9677	0.8961
ResUNet++	0.8307	0.7339	0.8692	**0.9765**	0.9197
UCTransNet	0.8178	0.7139	0.8533	0.9731	0.9093
Ours(supervised)	0.8339	0.7344	0.8544	0.9753	0.9109
Ours(hybrid self-supervised-supervised)	**0.8467**	**0.7461**	0.8725	0.9762	0.9214

From the results in Table 1, it is clear that the ResDDSCUNet++ model, combining self-supervised and supervised training, outperforms all other models across all metrics. Specifically, it surpasses the best-performing ResUNet++ by 1.6% in the Dice coefficient and 1.22% in the IoU coefficient. Although SegNet achieves the highest precision (88.77%) and F1 (92.47%), the proposed model follows closely with 87.25% and 92.14%. For recall, ResUNet++ leads with 97.65%, while the proposed model is very close at 97.62%. UNet shows the lowest recall score (96.14%), with a 1.51% difference. These results clearly demonstrate that our model, which integrates self-supervised and supervised training, significantly enhances feature extraction, outperforming other models in all aspects.

**Fig. 9:**
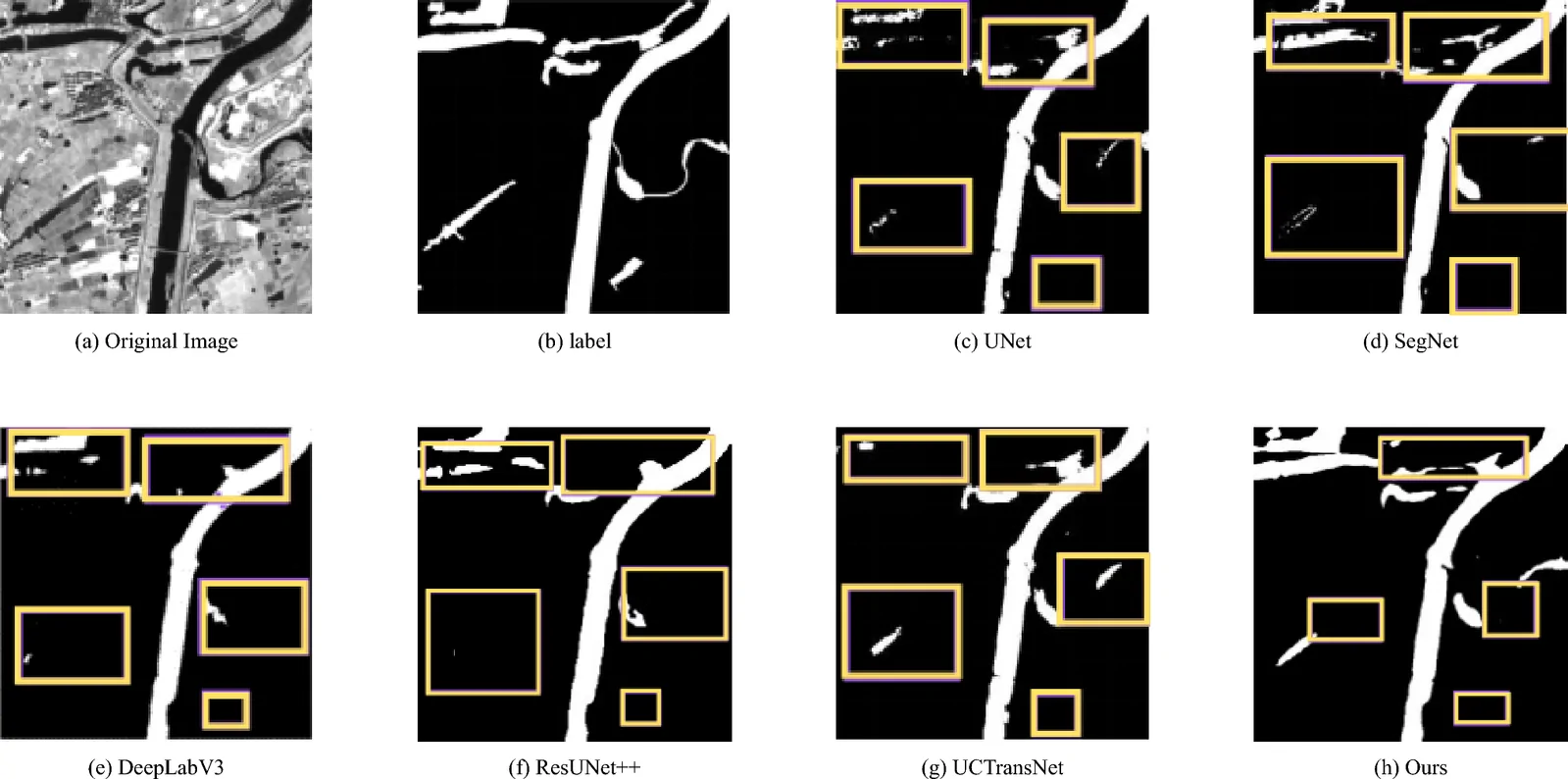
Visualization of segmentation results by different networks. The original image is sourced from the open-source water body segmentation dataset provided by the Baidu AI Open Platform^31^, featuring multispectral remote sensing images supplied by the Gaofen-2 (GF-2) satellite.

To more visually observe the performance differences between the method proposed in this paper and other image segmentation comparison methods, we conducted a visual comparison as shown in Fig. 9 below.

Figure 9 presents a complex mixed scenario containing main rivers, multiple small ponds, cross-river bridges, and building shadows, which is one of the most representative challenging scenarios in remote sensing water body extraction. We conduct a detailed region-by-region comparative analysis of the performance of each model in different areas of this scenario. In the main river area, UNet and SegNet produce obvious missegmentation at the boundary between rivers and riparian vegetation, misclassifying part of the vegetation as water bodies. Although DeepLabV3 improves the vegetation misdetection problem, it incorrectly truncates the river at the bridge position, destroying the continuity of the water body. ResUNet++ and UCTransNet can well identify the main body of the river, but their boundaries at river bends and irregular shorelines show obvious jaggedness and are not smooth enough. In the small water body area, UNet, SegNet, and DeepLabV3 all miss multiple isolated small ponds. ResUNet++ and UCTransNet can detect some small ponds but still have significant omissions, while our method can detect the vast majority of visible small water bodies in the scenario. In the shadow interference area, UNet, SegNet, and DeepLabV3 misclassify large areas of building shadows as water bodies. UCTransNet and ResUNet++ have improved shadow suppression effects but still have a certain degree of false detection. In contrast, our method can effectively distinguish shadows from real water bodies through the attention modulation mechanism, significantly reducing missegmentation caused by shadows. In terms of boundary accuracy, the water body boundaries of all comparison models have varying degrees of roughness and deviation, while the water body boundaries generated by our method are the smoothest and most accurate, with the highest degree of fit to the real shoreline. The visual comparison of this scenario clearly demonstrates that our method significantly outperforms other comparison models in three core dimensions: complex background interference resistance, small water body detection capability, and boundary fineness.

### Ablation experiments

In the ablation experiments, the effectiveness of each improved module within the proposed remote sensing image water body segmentation model, ResDDSCUNet++, was tested. This was conducted using the same dataset and consistent training parameters. The results are shown in Table 2 below.

**Table 2:** Evaluation results of UNet++ model after incorporating various modules.

Model	Dice	IoU	Precision	Recall	F1
UNet++	0.8118	0.7030	0.8429	0.9724	0.9030
UNeXt++	0.8190	0.7140	0.8523	0.9729	0.9086
AMM_UNeXt++	0.8256	0.7210	**0.8584**	0.9734	**0.9123**
DDSC_UNeXt++	0.8200	0.7142	0.8421	0.9730	0.9028
ResDDSC_UNeXt++	**0.8339**	**0.7344**	0.8544	**0.9753**	0.9109

Replacing the DoubleConv Block in the UNet++ network with the ConvNextV2 Block in the UNeXt++ model led to improvements of 0.72% in the Dice coefficient, 1.1% in the IoU coefficient, 0.94% in precision, 0.05% in recall, and 0.56% in F1. Adding the AMM after each ConvNextV2 Block in the AMM_UNeXt++ model resulted in further improvements: 0.66% in the Dice coefficient, 0.7% in the IoU coefficient, 0.61% in precision, 0.05% in recall, and 0.37% in F1. Although the AMM module enhanced performance, it also increased the network’s parameter count and computational load. According to data from TABLE 3, by incorporating the ConvNeXtV2 Block, the model’s parameter count was reduced by 0.727M, and the computational load decreased by 4.239G. Additionally, the training time per epoch was reduced by 1 minute, a 10% decrease, while the prediction time per image was shortened by 0.1 seconds, a reduction of 14%. These results convincingly demonstrate that introducing the ConvNeXtV2 Block, which features depthwise separable convolutions, can effectively reduce the parameter and computational burdens associated with large convolutional layers. However, when the AMM is added to the model, the parameter count increases by 3.05M, and the computational load rises by 5.886G. This change leads to a 44% increase in training time per epoch and a 33% increase in prediction time per image. These data indicate that although the AMM can enhance the network’s processing capabilities, it also adds to the model’s complexity and computational load. To reduce the network’s parameter count and computational demands, the model introduced a depthwise separable double convolution structure, DoubleDSC, which replaced the basic double convolution module, DoubleConv Block, in the model’s decoder. Experiments found that the model’s performance decreased, with the Dice coefficient, IoU coefficient, precision, recall, and F1 dropping by 0.56%, 0.68%, 1.63%, 0.04%, and 0.95% respectively. To mitigate the impact of the AMM module on network performance, the DoubleDSC structure was further improved by incorporating a residual connection, resulting in the ResDoubleDSC structure. As shown in TABLE 2, the inclusion of this structure enhanced network performance, with the Dice coefficient, IoU coefficient, precision, recall, and F1 reaching 83.39%, 73.44%, 85.44%, 97.53%, and 91.09% respectively. Compared to DDSC_UNet++, the Dice coefficient increased by 1.39%, the IoU coefficient by 2.02%, precision by 1.23%, recall by 0.23%, and F1 by 0.81%. This improvement demonstrates that the introduction of the ResDoubleDSC structure not only alleviates the performance decline caused by the depthwise separable convolutions but also significantly enhances the model’s feature extraction capabilities. Compared to AMM_UNeXt++, there was a significant reduction in both parameter count and computational load, as shown in Table 3, decreasing by 2.878M and 84.687G respectively. Additionally, the training time per epoch and the prediction time per image were reduced by 38% and 25%, respectively. The introduction of the ResDoubleDSC structure not only mitigated the performance decline associated with depthwise separable convolutions but also enhanced the model’s feature extraction capabilities.

**Table 3:** Comparison of model’s parameters, computational load, training time, and single image prediction time after introducing different modules.

Model	Parameters /M	FLOPs /G	TrainingTime (min/epoch)	PredictingTime (second/image)
UNet++	9.164	139.689	10	0.7
UNext++	**8.437**	135.450	9	**0.6**
AMM_ UNeXt ++	11.487	141.336	13	0.8
ResDDSC _UNeXt ++	8.609	**56.649**	**8**	**0.6**

From the experimental results, the introduction of the ConvNextV2 Block, AMM, and ResDoubleDSC modules has each enhanced the network’s ability to extract water body features to some extent. Compared to the classical UNet++ model, the Dice coefficient has improved by 2.21%, the IoU coefficient by 3.14%, precision by 1.15%, recall by 0.29%, and F1 by 0.79%, effectively enhancing the model’s capability to extract water body features.

**Fig. 10:**
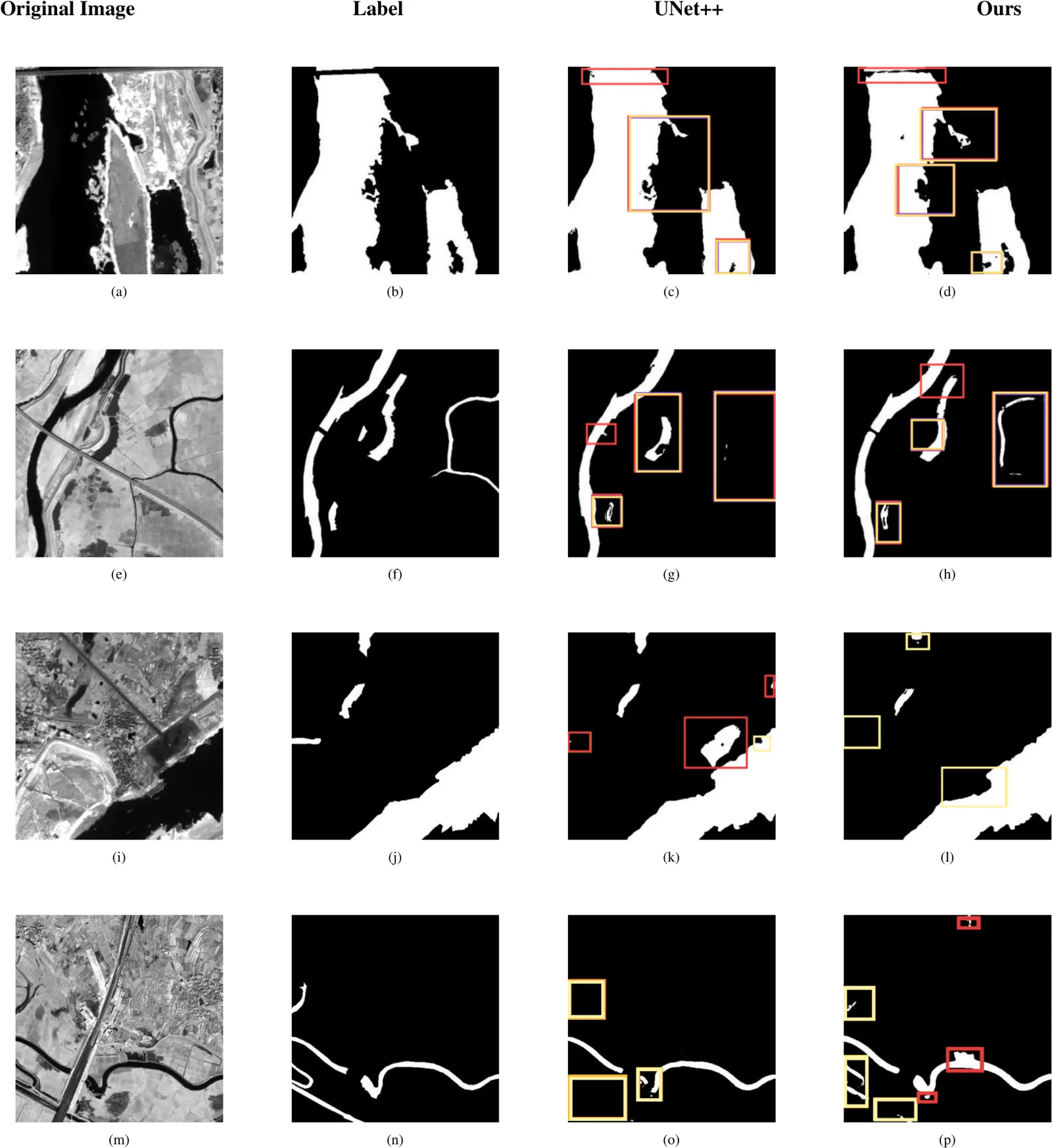
Water body segmentation examples of classical UNet++ network and ResDDSCUNet++ network. The original image is sourced from the open-source water body segmentation dataset provided by the Baidu AI Open Platform^31^, featuring multispectral remote sensing images supplied by the Gaofen-2 (GF-2) satellite.

Figure 10 shows four representative challenging scenarios to further compare the fine-grained performance differences between the classical UNet++ and our ResDDSCUNet++ model. The first scenario (a-d) depicts a meandering river with bridges. The classical UNet++ missegments most of the bridge deck area as water bodies, destroying the spatial continuity of the river. Meanwhile, it produces rough jagged boundaries at the junctions between rivers and farmland or vegetation, with obvious deviations from the real shoreline. In contrast, our method can accurately identify the entire bridge structure as non-water entities, completely preserving the natural form and topological structure of the river, and achieves smooth and accurate water body boundary delineation at the junctions of various ground objects. The second scenario (e-h) presents dense ponds with shadow interference. The classical UNet++ misses multiple small-sized ponds and misclassifies some large building shadow areas as water bodies, with obvious missed and false detection problems. In comparison, our method can detect the vast majority of small ponds in the scenario, only missing a very few extremely faint tiny water bodies, and has a strong suppression ability for building shadows with almost no misjudgment of shadows as water bodies, significantly improving the segmentation robustness in complex scenarios. The third scenario (i-l) features a complex scene covered by large-area building shadows. The classical UNet++ incorrectly segments a large contiguous dark building shadow area as water bodies, resulting in severe large-scale false detection. In contrast, our method effectively suppresses building shadow interference, with almost no false classification of shadow regions, demonstrating excellent spectral feature discrimination capability. The fourth scenario (m-p) illustrates a suburban scene with complex road networks and scattered tiny water bodies. The classical UNet++ misidentifies linear road segments as water bodies and fails to detect several extremely small water bodies, showing obvious limitations in distinguishing linear ground objects and capturing fine-grained water features. In comparison, our method accurately distinguishes roads from water bodies, correctly extracts most scattered tiny water bodies, and maintains clear and complete water body boundaries. The comparison results of these four scenarios consistently verify the synergistic effect of the three improved modules: the ConvNextV2 Block enhances global context perception to avoid the loss of large-scale water body features, the AMM focuses on easily overlooked small water bodies and edge regions, and the ResDoubleDSC module preserves fine-grained spatial details for precise boundary delineation.

The experiments demonstrate that the ResDDSC_UNext++ model can clearly extract most water bodies, including small bodies of water, and effectively distinguish details such as bridges over rivers. Additionally, the model has the capability to recognize commonly misidentified areas such as shadows. Compared to the classical UNet++, this model shows significant improvement in water body feature extraction, with a noticeable increase in extraction precision. After improving the Barlow Twins method, we designed three training approaches to test the effects of different training modes on water body segmentation. In the randomly initialized supervised training, 500 labeled images were used for training. In the hybrid training of self-supervised and supervised learning, each batch required one labeled image and two unlabeled images, necessitating the addition of 1000 unlabeled images. In the overall model training described in this paper, labeled images also participated in self-supervised training, thus effectively involving 2000 images in training. Below are the water body segmentation evaluation results for the three methods: randomly initialized training (500 labeled images), self-supervised initialized training (1500 unlabeled images, 500 labeled images), and combined self-supervised and supervised training (1000 unlabeled images, 500 labeled images), as shown in Table 4:

**Table 4:** Quantitative evaluation results of three training modes.

Method	Dice	IoU	Precision	Recall	F1
Supervised training random initialization	0.8339	0.7344	0.8544	0.9753	0.9109
Self-supervised initialization+ supervised training fine-tuning	0.8365	0.7450	0.8612	0.9758	0.9149
Mixed self-supervised and supervised training fine-tuning	**0.8467**	**0.7461**	**0.8725**	**0.9762**	**0.9214**

According to the data shown in Table 4, we can summarize the trend in overall accuracy changes: the purely supervised method with random initialization performed the worst, while the approach combining “self-supervised training with supervised fine-tuning” showed better results, ranking in the middle. The best performance was achieved by the strategy of “combined self-supervised and supervised training fine-tuning.” The performance metrics for this best-performing method—Dice coefficient, IoU coefficient, Precision, Recall, and F1—were 84.75%, 74.86%, 87.25%, 97.62%, and 92.14%, respectively, which represent improvements of 1.02%, 0.11%, 1.81%, 0.09%, and 1.05% over the randomly initialized supervised training. These results align with our expectations and further validate the effectiveness of the self-supervised learning paradigm. Clearly, self-supervised learning can extract universally beneficial knowledge from unlabeled imagery for downstream tasks.

This paper further explores the impact of using different numbers of unlabeled training images for self-supervised learning and using the obtained parameters to initialize the ResDDSCUNet++ network on its performance in segmentation tasks. The experimental results are shown in the following Table 5.

**Table 5:** Self-supervised segmentation evaluation results with different numbers of training images.

Number of self-supervised	Dice	IoU	Precision	Recall	F1
Training images					
1000	0.8345	0.7359	0.8544	0.9753	0.9109
1500	0.8365	0.7450	0.8612	0.9758	0.9149
4000	**0.8413**	**0.7526**	**0.8652**	**0.9762**	**0.9174**

Based on the data analysis from Table 5, we can draw the following conclusions: As the size of the dataset increases, the model’s performance metrics, such as the Dice coefficient and IoU coefficient, show an upward trend. However, when the number of self-supervised training images is only 1000 or 1500, the performance improvements in downstream segmentation tasks are not significant, with a Dice coefficient difference of 0.2%, IoU coefficient difference of 0.91%, precision difference of 0.68%, recall difference of 0.05%, and F1 difference of 0.4%. This phenomenon could be due to the fact that the magnitude difference between the supervised and self-supervised training data is not significant enough for the model to extract more effective generalization features. Nevertheless, when the number of images increases to 4000, the model’s ability to extract water body features is enhanced. The data shows that the Dice coefficient, IoU coefficient, precision, recall, and F1 are 84.13%, 75.26%, 86.52%, 97.62%, and 91.74%, respectively, representing increases of 0.68%, 1.67%, 1.08%, 0.09%, and 0.65% compared to training with only 1000 unlabeled images. This significant improvement in performance in downstream segmentation tasks indicates that an increase in data volume is beneficial for the self-supervised learning model’s feature extraction capability, especially when the data volume reaches a certain threshold, making the improvement more pronounced.

## Discussion

This study proposed an improved UNet++ architecture ResDDSCUNet++ for remote sensing water body extraction, which adopts ConvNextV2 block, introduces attention modulation module and ResDoubleDSC. The experimental results show that these improved modules can extract water segmentation accuracy. Specifically, The ConvNextV2 block can gain broader image information and higher-level abstract features and. enhance the capture of long-range dependencies. AMM can emphasize less significant regions to directly extract areas that are easy to overlook. This is crucial for segmentation tasks, as it helps the model focus on details that might be ignored but are significantly important for the final result. ResDoubleDSC structure not only alleviates the performance decline caused by the depthwise separable convolutions but also significantly enhances the model’s feature extraction capabilities.

In addition, we also found that a hybrid training method combining supervised learning and self-supervised learning can improve the accuracy of remote sensing water body extraction. This method fully utilizes unlabeled data related to the target dataset for pre training and fine-tuning the model. The experimental results in Table 4 also show that the hybrid self-supervised-supervised training method can significantly improve the accuracy of the model, with a 1.28% increase in Dice coefficient and a 1.17% increase in IoU compared to purely supervised training. Table 3 shows the impact of different modules on the number of parameters and computational complexity of the model, indicating that the ResDoubleDSC module greatly reduces the computational complexity while maintaining performance. This training method is a universal training approach that can serve as a reference and inspiration for training deep learning models for other tasks.

Extensive qualitative and quantitative results across all test scenarios demonstrate the strong generalization ability of our model. It maintains stable performance not only in simple scenes with single water body types and clear backgrounds but also in complex scenes with diverse water body morphologies, severe shadow interference, and dense ground object occlusion. This generalization capability stems from three aspects: (1) The ConvNextV2 Block learns robust global feature representations that are not sensitive to local noise; (2) The AMM module adaptively focuses on task-relevant features, reducing the impact of background interference; (3) The hybrid self-supervised-supervised training strategy learns general water body characteristics from a large amount of unlabeled data, enabling the model to adapt to unseen water body types and scenes.

Despite the advancements made in this study, several limitations remain. (1) Although our model utilizes the ConvNextV2 module to solve long-distance dependency problems. However, we did not adopt the recently proposed Mamba structure with stronger ability to extract global semantic information. While the ConvNextV2 block effectively enhances the model’s ability to capture long-range dependencies, the recently proposed Mamba structure demonstrates superior capabilities in extracting global semantic information. Given the focus of this study on improving the UNet++ architecture, integration of the Mamba structure was not explored here. Future work will investigate incorporating advanced architectures like Mamba to further optimize the model’s feature modeling for complex remote sensing scenes, particularly for global semantic correlation analysis in large-scale water bodies. Although the self-built dataset in this study covers various water body types, its scale and remote sensing modalities (e.g., SAR, hyperspectral data) are limited. Future research will validate the model’s generalizability on larger public datasets (such as the Gaofen-3 SAR dataset and Landsat multispectral data) and explore joint training with cross-modal remote sensing data to improve the model’s robustness across different resolutions and imaging conditions. While the hybrid training method improves model performance by leveraging unlabeled data, acquiring high-quality annotated data still requires significant human effort. Future studies will combine unsupervised learning (e.g., self-distillation, generative modeling) to further reduce dependency on annotated data, exploring semi-supervised or unsupervised frameworks for water body extraction to enhance practical deployment feasibility. (2) All experiments in this work were performed on the above curated Gaofen-2 optical multispectral public dataset. To the best of our knowledge after extensive literature review, no publicly available water body segmentation benchmark containing both BGR visible and NIR near-infrared four bands has been reported to date. Consequently, cross-dataset validation was not conducted in this study, which is a limitation and a core future research priority. Given the diversity of remote sensing data modalities and resolutions, future work will validate the proposed model on benchmarks covering different modalities (e.g., SAR, hyperspectral) and resolutions to evaluate its cross-modal and cross-resolution generalization. (3) Although we use a hybrid training method combining supervised learning and self-supervised learning, it still requires a large amount of manpower to annotate a large number of training samples.

While YOLO variants (e.g., YOLOv10 segmentation) excel in object detection, they are not optimized for pixel-level semantic segmentation tasks like water body extraction. UNet-based architectures, with their encoder-decoder structure and skip connections, are more suited for preserving spatial details critical for precise boundary delineation, as demonstrated in our experiments.

To address these limitations, future research can focus on several key areas. Firstly, we will attempt to introduce advanced Mamba structures into our model to further enhance the accuracy of remote sensing water segmentation. Secondly, the rapid advancement of remote sensing technology has significantly enhanced our capability to acquire diverse types of remote sensing data, thereby providing essential resources for efficient and precise water body detection. Modern remote sensing encompasses multiple modalities, including Synthetic Aperture Radar (SAR) and hyperspectral imaging, each offering unique advantages. Specifically, SAR technology provides all-weather, day-and-night imaging capabilities, which are particularly valuable for continuous monitoring applications. In our future work, we plan to rigorously evaluate the accuracy and robustness of our proposed model by conducting extensive experiments on larger-scale and more diverse remote sensing datasets, incorporating these multi-modal data sources. Finally, we will also attempt to combine self-supervised and unsupervised training methods to reduce the need for annotated training samples.

In addition, the recently proposed 3D neighborhood cross-differencing method has shown strong detail extraction capability in remote sensing image change detection. Future work will draw on the ideas of this method to further optimize the feature difference and detail enhancement modules of the model and improve the segmentation accuracy of water body boundaries.

This study presents ResDDSC-UNet++, an enhanced UNet++ architecture for remote sensing water body extraction, which integrates three key innovations: the ConvNextV2 block, an attention modulation module (AMM), and a ResDoubleDSC structure. The experimental results demonstrate that these improvements collectively enhance water segmentation accuracy, addressing several critical challenges in remote sensing image analysis.

The ConvNextV2 block can gain broader image information and higher-level abstract features and. enhance the capture of long-range dependencies. The AMM plays a pivotal role in emphasizing less prominent regions, enabling the model to accurately extract areas that are frequently overlooked. This capability is particularly crucial for segmentation tasks, as it directs the model’s attention to fine-grained details that are often neglected but essential for achieving high-precision results. Furthermore, the ResDoubleDSC structure effectively mitigates the performance degradation typically associated with depthwise separable convolutions while simultaneously enhancing the model’s feature extraction capabilities. This dual benefit ensures robust feature representation, which is vital for handling the complex and heterogeneous nature of remote sensing imagery.

In addition to architectural improvements, we propose a hybrid training strategy that combines supervised learning with self-supervised learning. This approach leverages both labeled and unlabeled data, utilizing domain-relevant unlabeled datasets for pretraining followed by task-specific fine-tuning. As evidenced by the results in Table 4, this hybrid training method significantly improves model accuracy, achieving a 1.17% increase in IoU compared to traditional supervised learning alone. The success of this training paradigm suggests its potential as a generalizable framework for other remote sensing tasks, particularly in scenarios where labeled data is scarce but unlabeled data is abundant.

Despite these advancements, several limitations warrant further investigation. First, while the ConvNextV2 module effectively addresses long-range dependency issues, we did not explore more recent architectures such as the Mamba structure, which has demonstrated superior capabilities in extracting global semantic information. Incorporating such advancements could further enhance the model’s performance, particularly for large-scale remote sensing scenes. Second, our experiments were conducted on a custom dataset with limited scale and diversity. Given the variability in remote sensing image types and resolutions, future work should validate the model on larger and more diverse datasets, such as [specific benchmark datasets], to ensure its generalizability across different imaging conditions. Third, although the hybrid training method shows promise, its effectiveness depends on the quality and relevance of the unlabeled data used for pretraining. Future research should investigate strategies to optimize the selection and utilization of unlabeled data, as well as explore more advanced self-supervised learning techniques tailored to remote sensing applications.

In conclusion, ResDDSC-UNet++ represents a significant step forward in remote sensing water body extraction, offering both architectural and methodological innovations. However, addressing the identified limitations could further enhance its performance and applicability, paving the way for more robust and generalizable solutions in the field of remote sensing image analysis.

This study proposes ResDDSC-UNet++, an enhanced UNet++ architecture for remote sensing water body extraction, which integrates three key innovations: the ConvNextV2 block, an attention modulation module (AMM), and a ResDoubleDSC structure. The experimental results demonstrate that these improvements collectively enhance water segmentation accuracy, addressing several critical challenges in remote sensing image analysis.

The ConvNextV2 block significantly improves the model’s ability to capture broader image information and higher-level abstract features, particularly enhancing its capacity to model long-range dependencies. This is crucial for accurately delineating water bodies in complex remote sensing scenes. The AMM further refines the segmentation process by emphasizing less prominent regions, enabling the model to accurately extract areas that are frequently overlooked. This capability is particularly valuable in segmentation tasks, as it directs the model’s attention to fine-grained details that are often neglected but essential for achieving high-precision results. Additionally, the ResDoubleDSC structure effectively mitigates the performance degradation typically associated with depthwise separable convolutions while simultaneously enhancing the model’s feature extraction capabilities. This dual benefit ensures robust feature representation, which is vital for handling the complex and heterogeneous nature of remote sensing imagery.

In addition, we propose a hybrid training strategy that combines supervised learning with self-supervised learning. This approach leverages both labeled and unlabeled data, utilizing domain-relevant unlabeled datasets for pretraining followed by task-specific fine-tuning. The experimental results presented in Table 4 demonstrate the efficacy of the hybrid training method, with the model achieving notable improvements of 1.28% in Dice coefficient and 1.17% in IoU compared to conventional supervised learning approaches. These quantitative enhancements underscore the effectiveness of combining supervised and self-supervised learning paradigms for remote sensing water body extraction tasks. The success of this training paradigm suggests its potential as a generalizable framework for other remote sensing tasks, particularly in scenarios where labeled data is scarce but unlabeled data is abundant.

Despite these advancements, several limitations warrant further investigation. First, while the ConvNextV2 module effectively addresses long-range dependency issues, we did not explore more recent architectures such as the Mamba structure, which has demonstrated superior capabilities in extracting global semantic information. Incorporating such advancements could further enhance the model’s performance, particularly for large-scale remote sensing scenes. Second, the proposed model has been validated using the Gaofen-2 optical multispectral dataset. Given the variability in remote sensing data modalities and imaging resolutions, future work will first prioritize constructing and curating more four-band water body segmentation datasets to enable standardized cross-dataset evaluation. We will then extend validation to larger, more diverse benchmarks including Gaofen-3 SAR and Landsat multispectral datasets, to comprehensively assess the model’s generalizability and robustness across different imaging conditions. Third, although the hybrid training method shows promise, it still requires substantial human effort to annotate a large number of training samples, which remains a bottleneck for scalability.

To address these limitations, future research will focus on several key directions. First, we plan to integrate advanced Mamba structures into our model to further enhance its ability to capture global semantic information and improve segmentation accuracy. Second, we will validate the model’s performance on larger and more diverse remote sensing image datasets to ensure its robustness across different scenarios. Finally, we aim to explore the combination of self-supervised and unsupervised training methods to reduce the reliance on annotated training samples, thereby improving the scalability and practicality of the approach.

## Conclusion

In this paper, we addressed the issue of limited receptive fields in deep learning-based water body extraction methods by enhancing the UNet++ network structure. The proposed purely supervised version of ResDDSCUNet++, which integrates the ConvNeXtV2 module, Attention Modulation Module (AMM), and residual depthwise separable double convolution (ResDoubleDSC) module, achieved a Dice coefficient of 83.39% and an IoU of 73.44%, representing improvements of 2.21% and 3.14% respectively compared to the baseline UNet++ model. On this basis, after introducing the hybrid self-supervised-supervised training strategy, the model performance was further improved to Dice=0.8467 and IoU=0.7461, representing increases of 1.28% and 1.17% respectively compared to the purely supervised version. These quantitative results demonstrate that ResDDSCUNet++ can more accurately extract water bodies—even small or lightly colored ones—and delineate their boundaries with high precision. Future work will extend these experiments to additional remote sensing datasets and further optimize the network architecture.

## Data Availability

The data that supports the findings of this study is available from the corresponding author upon reasonable request.
